# Anaphylatoxin signaling activates macrophages to control intracellular *Rickettsia* proliferation

**DOI:** 10.1128/spectrum.02538-23

**Published:** 2023-10-19

**Authors:** Mustapha Dahmani, Jinyi C. Zhu, Jack H. Cook, Sean P. Riley

**Affiliations:** 1 Department of Veterinary Medicine, University of Maryland-College Park, College Park, Maryland, USA; 2 Virginia-Maryland College of Veterinary Medicine, College Park, Maryland, USA; University of North Dakota, Grand Forks, North Dakota, USA

**Keywords:** anaphylatoxin, complement system, intracellular bacteria, *Rickettsia*, macrophage

## Abstract

Pathogenic *Rickettsia* species proliferate within the cytoplasm of permissive host cells *in vivo*. The cytoplasm of these host cells is adequate to support the complex metabolic and physiological needs for *Rickettsia* growth. However, a dramatic host/pathogen interplay occurs when *Rickettsia* encounter innate immune cells, whereby the bacteria can proliferate as normal or the host can restrict bacterial growth. This interplay is most divergent within myeloid host cells, where intra- and extracellular factors can produce either successful *Rickettsia* parasitism or innate immune control of bacterial proliferation. With the prior knowledge that the mammalian complement system is activated during mammalian infection, we sought to determine if extracellular complement activation and anaphylatoxin signaling can modify the fate of *Rickettsia* within mononuclear host cells. Results indicate that supplementation of growth media with either C3a or C5a anaphylatoxin peptides is sufficient for many myeloid cells to control the proliferation of multiple different *Rickettsia* species. Chemical or genetic disruption of anaphylatoxin signaling or anaphylatoxin receptors eliminates complement-induced restriction of bacterial proliferation. Finally, anaphylatoxin signaling modifies macrophage physiology by inducing inflammatory phenotypes that ultimately control the intracellular proliferation of these pathogens.

**IMPORTANCE:**

Pathogenic *Rickettsia* species are extremely dangerous bacteria that grow within the cytoplasm of host mammalian cells. In most cases, these bacteria are able to overpower the host cell and grow within the protected environment of the cytoplasm. However, a dramatic conflict occurs when *Rickettsia* encounter innate immune cells; the bacteria can “win” by taking over the host, or the bacteria can “lose” if the host cell efficiently fights the infection. This manuscript examines how the immune complement system is able to detect the presence of *Rickettsia* and alert nearby cells. Byproducts of complement activation called anaphylatoxins are signals that “activate” innate immune cells to mount an aggressive defensive strategy. This study enhances our collective understanding of the innate immune reaction to intracellular bacteria and will contribute to future efforts at controlling these dangerous infections.

## INTRODUCTION

Pathogenic species of the genus *Rickettsia* are the causative agents of life-threatening arthropod-transmitted infections. While historically *Rickettsia* species have been transmitted by many different arthropods, the most persistently endemic pathogens are transmitted by tick vectors. Human case frequency correlates with the geographic distribution and behavior of these vector arthropods (1 – 3). Cases of tick-borne spotted fever group *Rickettsia* infections increased by 23% from 2016 to 2018 within the USA, and additional rickettsial diseases are distributed throughout the world, with endemic and hyper-endemic areas across the globe (4). The most impactful species in the USA are *Rickettsia rickettsii*, the etiologic agent of Rocky Mountain spotted fever (RMSF), and *Rickettsia parkeri*, the cause of *Rickettsia parkeri* rickettsiosis (3). The case fatality rate for RMSF in the preantibiotic era was 20%–25% and remains at 3%–4% (5, 6).

*Rickettsia* species are Gram-negative obligate intracellular bacteria that proliferate within the cytosol of eukaryotic host cells (7). These organisms are maintained in nature through an endozoonotic cycle of parasitizing hematophagous arthropods and transmission to mammals during arthropod feeding (8, 9). After transmission to mammalian hosts, *Rickettsia* species primarily invade and proliferate within cells of the endothelial system. While infected endothelial cells detect the intracellular bacteria and respond accordingly, *Rickettsia* species are able to sufficiently manipulate host cell signaling and innate immune responses to generate a permissive growth environment (10 – 12). As the disease progresses, immune recognition of *Rickettsia* and endothelial cell signaling contribute to the recruitment of innate leukocytes (13). Subsequent encounters between mononuclear cells and *Rickettsia* produce vastly different outcomes, with either *Rickettsia* invading into and successfully colonizing the monocyte or the monocyte effectively controlling bacterial proliferation with associated proinflammatory signaling (14 – 19).

The complement system has traditionally been considered an arm of the innate immune system, whereby soluble proteins circulate through bodily fluids as inactive precursors waiting to be activated by innate immune sensors of infection (20, 21). The ensuing complement activation can directly temper infections by generating lytic pores on the surface of the pathogen, by coating the particle in complement opsonins that enhance uptake by innate immune cells, or by generating massively proinflammatory anaphylatoxin peptides (21). The complement system is activated directly by *Rickettsia in vitro* and during mammalian infection *in vivo* (22 – 25). However, the bacilli have evolved mechanisms to combat complement-mediated killing (26 – 28). While the antibacterial membrane attack complex and opsonization are dispensable for the immune response to *Rickettsia* infection, complete ablation of complement functionality drastically reduces the veracity of the immune response to infection (24). We therefore initiated this work with the knowledge that the complement system contributes to the effective immune response to infection but lacked knowledge of the molecular or cellular mechanisms of efficacy.

The overarching hypothesis that drove this investigation is that *Rickettsia*-induced complement activation generates proinflammatory anaphylatoxins and peptides that subsequently modulate leukocytes to control intracellular infection. The primary anaphylatoxins, C3a and C5a, are 70–80 amino acid peptides that bind to the anaphylatoxin chemotactic receptors C3a receptor (C3aR), C5a receptor 1 (CD88, C5aR1), and C5a receptor 2 (GPR77, C5aR2) (29 – 31). Interaction of soluble anaphylatoxins with cognate anaphylatoxin receptors induces vasodilation and activation of various immune cells (32 – 34). The classical anaphylatoxin receptors C3aR and C5aR1 are transmembrane G-protein-coupled receptors that detect extracellular anaphylatoxin peptides to activate intracellular heterotrimeric G-proteins. These G-proteins subsequently activate multiple signaling cascades, including adenylyl cyclases, phospholipase D, protein kinase C, PI3 kinases, and Ca^2+^ depolarization (35 – 42). The intracellular hallmarks of anaphylatoxin activation include extracellular signal-related kinase 1/2 (ERK1/2) phosphorylation and NF-κB activation with increased expression of proinflammatory cytokines, co-stimulatory molecules, and complement components (41, 43, 44).

In the present work, we have examined the effects of anaphylatoxins and anaphylatoxin receptors on *Rickettsia* intracellular growth within myeloid cells. We have developed different *in vivo* and *ex vivo* approaches to evaluate macrophage responses to anaphylatoxin stimuli by evaluating cellular responses and bacterial survival. Our findings advance our understanding of the immune response to *Rickettsia* infection by establishing the role of the anaphylatoxin-anaphylatoxin receptor axis in controlling *Rickettsia* proliferation.

## RESULTS

### Anaphylatoxin-activated RAW264.7 murine macrophage cells limit intracellular growth of pathogenic *Rickettsia*

Previous studies have demonstrated that genetic ablation of the murine complement system results in increased sensitivity to *Rickettsia australis* infection, with mortality occurring prior to the development of a fulminant adaptive immune response (24, 45). Therefore, *Rickettsia*-induced complement activation likely contributes to innate immune control of infection *in vivo*. Related molecular and cellular analyses of the direct interaction between pathogenic *Rickettsia* and the complement system have also indicated that these bacteria are not susceptible to either the antibacterial membrane attack complex or complement receptor-mediated phagocytosis, thus indirectly implicating the anaphylatoxin/anaphylatoxin receptor system as the arm of the complement system that contributes to the control of *Rickettsia* infection (24). We therefore sought to assess the hypothesis that anaphylatoxin signaling is essential for restricting *Rickettsia* growth within host cells.

**FIG 1:**
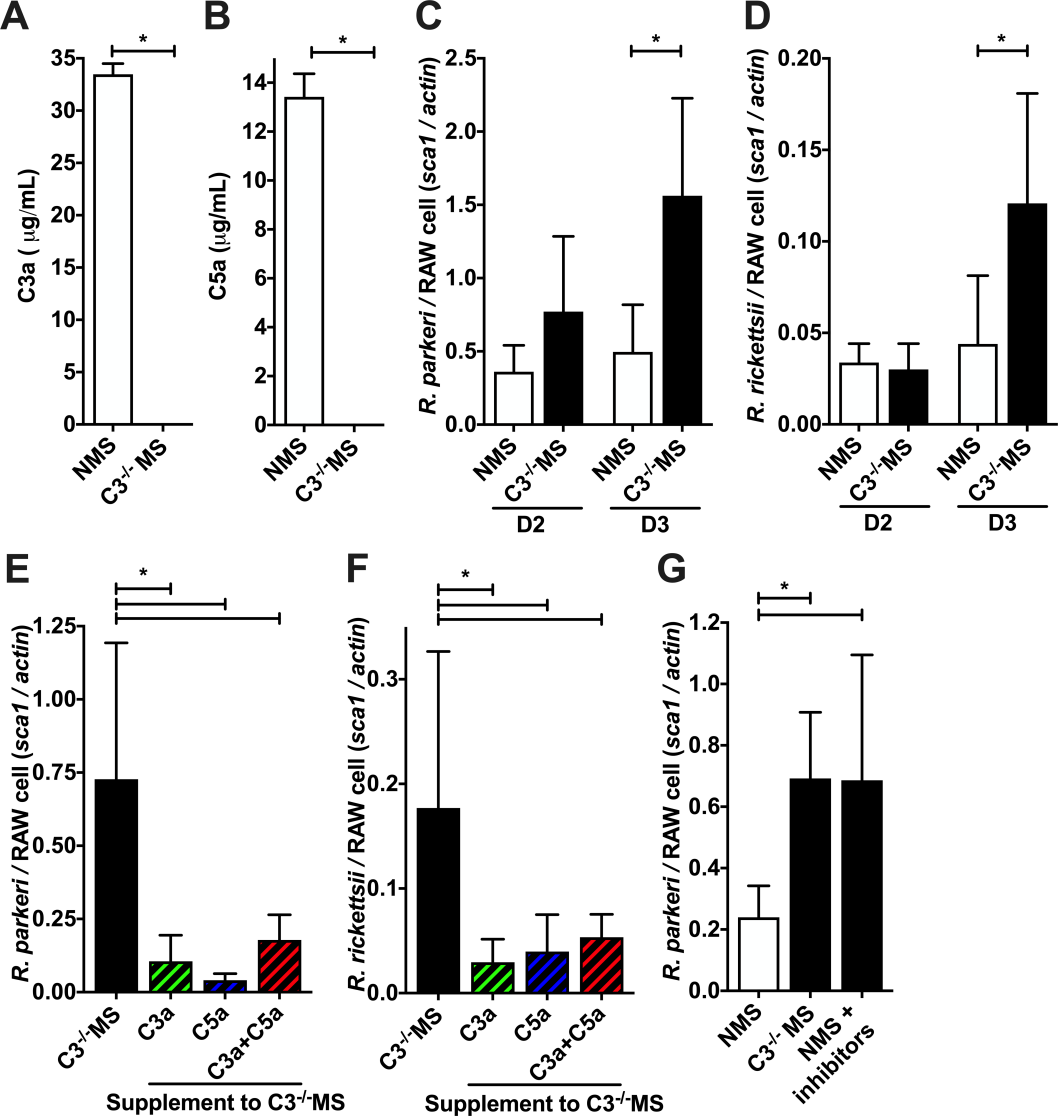
Murine anaphylatoxins reduce *Rickettsia* proliferation in RAW264.7 cells. (**A and B**) Presence of the anaphylatoxins C3a (**A**) and C5a (**B**) in the media after 3 days of culture of *R. parkeri*-infected RAW264.7 cells in the presence of NMS and C3^−/−^ MS, as determined by an enzyme-linked immunosorbent assay. (**C and D**) Proliferation of *R. parkeri* (**C**) and *R. rickettsii* (**D**) in RAW264.7 cells cultivated with complement-active serum (NMS) or complement-deficient serum (C3^−/−^ MS), as determined by the quantitative PCR ratio of *Rickettsia sca1* to murine *actin* DNA. (**E and F**) Quantity of *R. parkeri* (**E**) and *R. rickettsii* (**F**) after 3 days of culture in RAW264.7 cells with complement-deficient serum (C3^−/−^ MS) supplemented with phosphate-buffered saline, mouse C3a peptide, mouse C5a peptide, or both C3a and C5a peptides. (**G**) Presence of *R. parkeri* after 3 days of culture in RAW264.7 cells with complement-active serum (NMS), complement-deficient serum (C3^−/−^ MS), or complement-active serum with anaphylatoxin receptor antagonists PMX53 and SB290170 (NMS + inhibitors). *P < 0.05 by (A,B) Student’s *t*-test; (C,D) one-way ANOVA with Sidák’s multiple comparison of matched days; (E,F,G) one-way ANOVA with Dunnett’s multiple comparison test to the NMS/mock-treated control. All columns represent at least five data points, and experiments were repeated to ensure reproducibility.

The most direct method for eliminating complement activity from murine serum is to genetically ablate the central complement component C3 (46). While complement activation is readily inducible in normal mouse serum (NMS) during *Rickettsia* infection, C3-deficient mouse serum (C3^−/−^ MS) is incapable of generating complement effector mechanisms, including forming the membrane attack complex, opsonizing bacteria, or, importantly, producing the proinflammatory anaphylatoxin peptides C3a and C5a (24). As shown in Fig. 1A and B, both C3a and C5a are produced when *R. parkeri*-infected RAW264.7 cells are cultured for 3 days in media containing NMS. However, neither C3a nor C5a is produced when the same cells are grown in media containing C3^−/−^ MS, demonstrating that C3^−/−^ MS does not stimulate C3a nor C5a anaphylatoxin production and therefore cannot induce signaling through the reciprocal anaphylatoxin receptors on mammalian cells.

The murine RAW264.7 cell line has previously been employed to investigate the host-pathogen interplay occurring within *Rickettsia*-infected macrophages (47, 48). RAW264.7 cells express the anaphylatoxin receptors on their surface and are capable of responding to extracellular anaphylatoxins (42, 49 – 52). To determine if complement influences *Rickettsia* proliferation in these macrophages, we infected RAW264.7 cells with the causative agent of *Rickettsia parkeri* rickettsiosis in the presence (NMS) or absence (C3^−/−^ MS) of functional complement. DNA was extracted at 2 and 3 days post infection to assess *R. parkeri* proliferation as determined by calculating the quantitative PCR (qPCR) ratio of *Rickettsia* DNA (*sca1*) to mouse DNA (*actin*). As shown in Fig. 1C, RAW264.7 cells cultured in media containing complement-active serum and complement-deficient C3^−/−^ serum did not produce differences in *R. parkeri* growth through 2 days of culture. However, there was significantly more *Rickettsia* at 3 days post infection when grown in the absence of complement as compared to complement-active NMS. It is also important to note that there is no statistical difference in the survival of RAW264.7 cells infected with *R. parkeri* and cultured in the presence of NMS or C3^−/−^ MS throughout the 3 days of culture (Fig. S1), so the changes in *Rickettsia* load are not associated with the death of the host cells. To validate this finding in other *Rickettsia*, we infected RAW264.7 cells with the causative agent of Rocky Mountain spotted fever, *R. rickettsii*, in the presence of complement-active NMS or complement-deficient C3^−/−^ MS. As shown in Fig. 1D, *R. rickettsii* also proliferated better in the absence of complement activation than in complement-active normal human serum (NHS). These results suggest that complement activation induces murine RAW264.7 macrophages to restrict the growth of *Rickettsia*.

To examine the potential that anaphylatoxin-independent complement activities were responsible for restricting *Rickettsia* growth*,* we performed *R. parkeri* infection in the absence of complement activation by using C3^−/−^ MS but supplementing the media with a physiologically relevant concentration of purified C3a and C5a peptides to reconstitute anaphylatoxin signaling (53). C3a and C5a are small polypeptides that act as potent inflammatory mediators targeting a broad spectrum of immune and nonimmune cells, including macrophages (54). *R. parkeri* and *R. rickettsii* were again cultured in RAW264.7 cells with C3^−/−^ MS supplemented with phosphate-buffered saline (PBS), C3a peptide, C5a peptide, or both C3a and C5a at 1 µg/mL. The addition of these peptides does not affect the survival of RAW264.7 cells (Fig. S2). Quantitative PCR of DNA extracted from the cells on day 3 demonstrates that anaphylatoxin supplementation significantly restricted *R. parkeri* and *R. rickettsii* growth as compared to permissive growth in C3^−/−^ MS (Fig. 1E and F). These data directly implicate C3a and C5a peptides in enhancing the restriction of *Rickettsia* growth.

The anaphylatoxin peptides C3a and C5a exert their inflammatory effects through interaction with specific anaphylatoxin receptors called C3aR, C5aR1, and C5aR2 (55). To determine if anaphylatoxin-induced restriction of *Rickettsia* growth is mediated by the anaphylatoxin-anaphylatoxin receptor axis, we cultivated RAW264.7 cells in complement-active NMS, complement-deficient C3^−/−^ MS, or complement-active NMS in the presence of the anaphylatoxin receptor inhibitors PMX53 and SB290170. SB290157 is an antagonist of C3aR (56), whereas PMX53 is a competitive antagonist of the C5aRs (57). After 3 days of growth in RAW264.7 cells, *R. parkeri* growth was restricted in complement-active media (NMS) as compared to complement-deficient media C3^−/−^ MS (Fig. 1G). However, in media with reconstituted complement activity (NMS), chemical inhibition of the anaphylatoxin receptors restores *R. parkeri* proliferation, demonstrating that restriction of *Rickettsia* growth in macrophages requires the anaphylatoxin receptors. Taken together, the data in Fig. 1 demonstrate that complement activity can restrict *Rickettsia* growth in murine RAW264.7 macrophages, that C3a or C5a is sufficient to decrease bacterial growth, and that signaling through the anaphylatoxin receptors is required for complement-mediated restriction of *Rickettsia* proliferation.

### Anaphylatoxin-mediated reduction of *Rickettsia* growth in human cells

**FIG 2:**
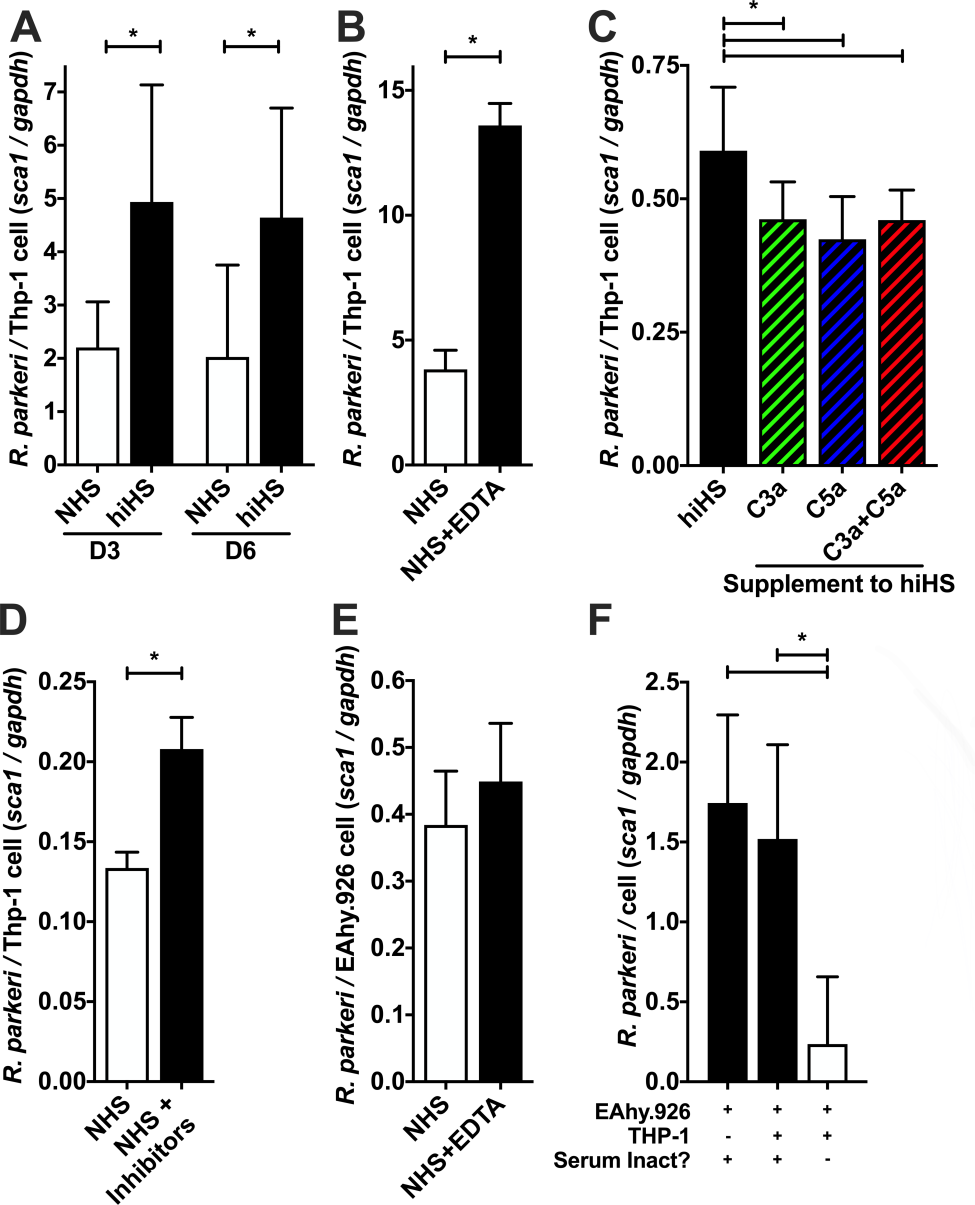
Human anaphylatoxins restrict *Rickettsia* proliferation in THP-1 cells. (**A**) *R. parkeri* proliferation in human THP-1 monocytes cultivated with either complement-active NHS or complement-deficient hiHS, as determined by the qPCR ratio of *Rickettsia sca1* to human *gapdh* DNA. (**B**) Growth of *R. parkeri* cultivated for 3 days in THP-1 cells with either NHS or complement-deficient EDTA-inactivated human serum. (**C**) Quantity of *R. parkeri* in THP-1 cells cultivated in complement hiHS supplemented with PBS, human C3a peptide, human C5a peptide, or both C3a and C5a peptides. (**D**) Quantity of *R. parkeri* after 3 days of growth in THP-1 cells cultivated in complement-active serum (NHS) or NMS plus the anaphylatoxin receptor antagonists PMX53 and SB290170 (NHS + inhibitors). (**E**) *R. parkeri* proliferation in EA.hy926 human endothelial cells cultivated complement-active NHS or complement-inactivated NHS + EDTA. (**F**) *R. parkeri* growth in endothelial (EA.hy926) and macrophage (THP-1) co-culture. *R. parkeri* proliferates in ea.hy926 or co-culture in complement-inactive serum, but growth is restricted in EA.hy926/THP-1 co-culture containing complement-active serum. *P < 0.05 by (A) one-way ANOVA with Sidák’s multiple comparison of matched days; (B,D,E) Student’s *t*-test; (C) one-way ANOVA with Dunnett’s multiple comparison test to mock-treated control; (F) one-way ANOVA with Tukey’s multiple comparison test of all columns. All columns represent at least five data points, and experiments were repeated to ensure reproducibility.

As multiple *Rickettsia* species are intracellular pathogens in humans, we sought to determine if the anaphylatoxin-anaphylatoxin receptor axis also supports the constraint of *Rickettsia* proliferation within human monocytic cells. To this end, we examined the influence of the anaphylatoxins on *R. parkeri* growth with human THP-1 cells. These macrophages are of particular relevance to *Rickettsia* as bacterial proliferation in these cells correlates with mammalian virulence (14, 58, 59), and there appears to be a dynamic interplay between the bacteria and host occurring within these cells (15, 60). We employed normal human serum to drive THP-1 differentiation/adherence in the absence of exogenous THP-1 activators like PMA. Also, in the absence of the ability to genetically ablate complement activity as with C3^−/−^ mouse serum, we instead generated complement-deficient human serum through physical heat inactivation (heat-inactivated human sera, hiHS) or chemical inhibition with ethylenediaminetetraacetic acid (NHS + EDTA) (61, 62). Notably, neither heat inactivation nor EDTA supplementation is toxic to THP-1 cells (Fig. S3 and S4). *R. parkeri*-infected THP-1 was cultured for 3 days in either NHS or hiHS. As shown in Fig. 2A, complement activity in the NHS reduces *R. parkeri* growth as compared to complement-inactivated hiHS. Subsequent chemical inhibition of complement activity (NHS + EDTA) also permitted greater proliferation in THP-1 cells than that of NHS (Fig. 2B). These data demonstrate that complement activity is capable of driving human THP-1 cells to limit the growth of *Rickettsia*.

To determine if anaphylatoxin stimulation is essential for complement-induced restriction of *Rickettsia* proliferation in THP-1 cells, we infected *R. parkeri* in the presence of human anaphylatoxin peptides. The complement system was nonfunctioning in hiHS, but the addition of anaphylatoxins is sufficient to decrease *Rickettsia* proliferation at 3 days post infection (Fig. 2C). Additionally, chemical inhibition of anaphylatoxin receptors eliminates the decreased *Rickettsia* proliferation induced by complement activation without affecting the health of the host cells (Fig. 2D and S5). Together, these data demonstrate that anaphylatoxin and anaphylatoxin receptor interactions restrict *R. parkeri* proliferation in human monocytic cells.

While monocytic cells are of particular interest for their differential interaction with pathogenic *Rickettsia*, these bacteria have a primary tropism for endothelial cells *in vivo* (13, 63, 64). To this end, we investigated the effect of complement activation on the growth of *R. parkeri* in EA.hy926 human endothelial cells. As shown in Fig. 2E, complement-active NHS is not sufficient to restrict *R. parkeri* growth in EA.hy926 at 3 days post infection as compared to complement-inhibited NHS + EDTA. This finding contrasts with the observed complement-mediated restriction of growth in murine and human monocytes (Fig. 1C and D; Fig. 2B).

This endothelial versus monocyte dichotomy highlights the possibility that anaphylatoxin-activated monocytes could secrete factors to induce endothelial cells to restrict *Rickettsia* proliferation. This concept is conceivable because exogenously activated endothelial cells have been demonstrated to directly reduce *Rickettsia* growth (13, 65, 66). To assess the validity of the hypothesis that anaphylatoxin-treated monocytes can indirectly control *Rickettsia* growth, we set up an EA.hy926 endothelial + THP-1 macrophage co-culture. As shown in Fig. 2F, there is no significant difference in *R. parkeri* growth at 3 days post infection within EA.hy926 or EA.hy926 + THP-1 co-culture, so long as the infected cells are cultivated in media lacking functional complement. However, *R. parkeri* proliferation is reduced in EA.hy926 + THP-1 co-culture when propagated in complement-active NHS; this demonstrates that complement activation is not sufficient to restrict bacterial growth in endothelial cells, but the addition of complement-activated macrophages is sufficient to modulate the growth environment to decrease *Rickettsia* proliferation.

### Anaphylatoxin-mediated restriction of *R. australis* proliferation in primary murine bone marrow macrophages

**FIG 3:**
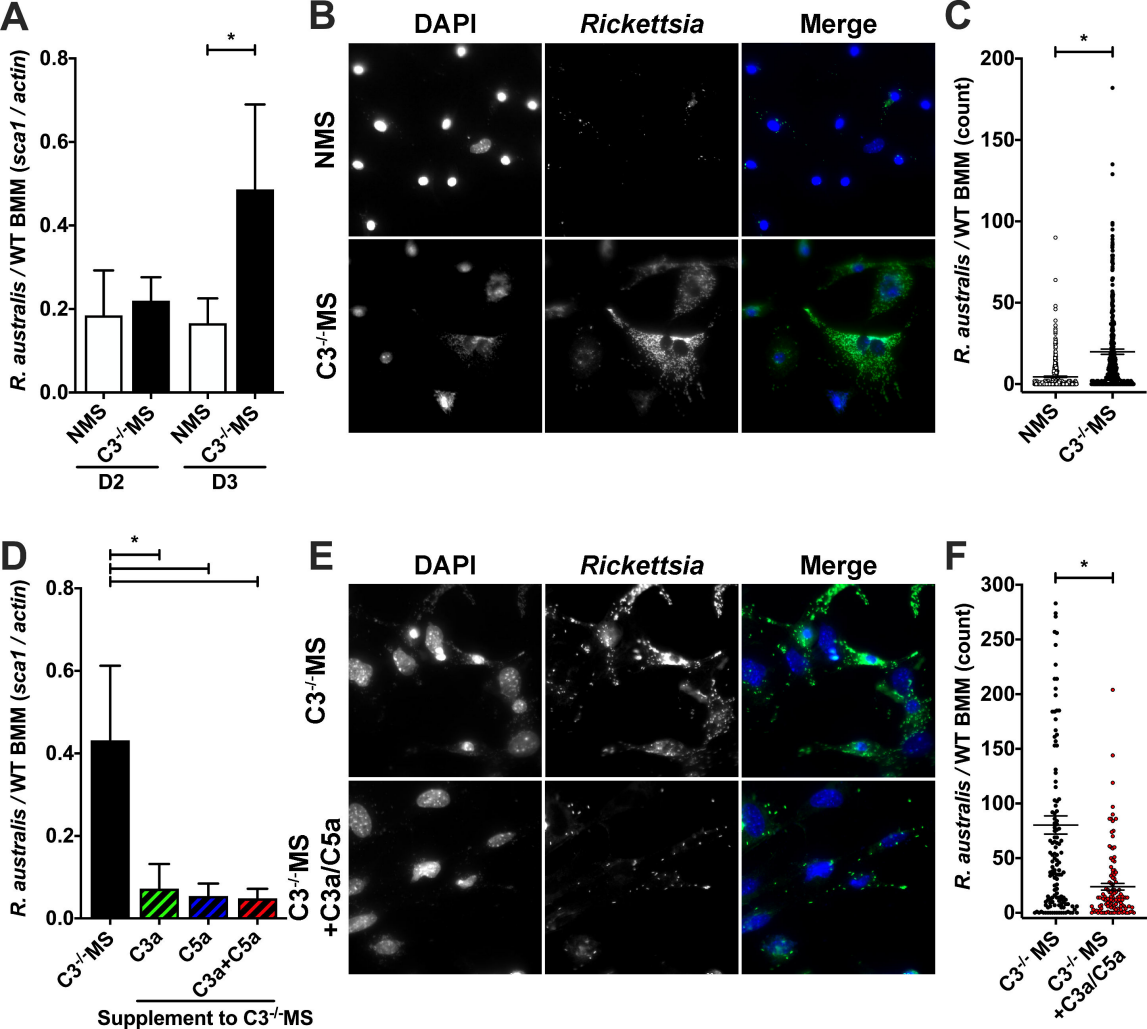
Anaphylatoxins restrict *Rickettsia australis* proliferation in C57BL/6J bone marrow macrophages. (**A, B, C**) *R. australis* proliferation in C57BL/6J BMMs cultured in complement-active NMS or complement-deficient C3^−/−^ MS as determined by (**A**) qPCR ratio of *R. australis sca1* to murine *actin*, (**B**) fluorescent microscopic imaging, and (**C**) visual quantification of *R. australis* and BMM nuclei in over 600 BMMs when cultured for 3 days in the presence of NMS or C3^−/−^ MS. (**D, E, F**) Quantity of *R. australis* after culture in BMM grown with complement-deficient C3^−/−^ MS supplemented with PBS, mouse C3a peptide, mouse C5a peptide, or both C3a and C5a peptides as determined by (**D**) qPCR, (**E**) fluorescence microscopy, and (**F**) visual quantification of *R. australis* in 600 BMMs. *P < 0.05 by (A) one-way ANOVA with Sidák’s multiple comparison of matched days; (C,F) Student’s *t*-test; (D) one-way ANOVA with Dunnett’s multiple comparison test to C3^−/−^ MS. Scale bar = 50 µm. All columns represent at least five data points, and experiments were repeated to ensure reproducibility. The scatter plot with mean and SEM represents at least 600 different individual cells.

Having demonstrated a role for anaphylatoxins and anaphylatoxin receptors within immortalized murine and human macrophage cell lines, we sought to further examine these phenotypes within primary cells. To this end, we isolated tibial and humeral bone marrow macrophages (BMMs) from C57BL/6J mice. We deduced that it was important to employ *R. australis* in this model because this is the sole *Rickettsia* species capable of causing disease in the murine B6 background (45). Macrophage colony-stimulating factor (M-CSF)-differentiated C57BL/6J BMMs were infected with *R. australis* in media containing complement-active NMS or complement-deficient C3^−/−^ MS. While *R. australis* propagated equally well in media containing NMS or C3^−/−^ MS through day 2 of propagation, there was a significant restriction of *R. australis* growth at day 3 of infection within BMMs treated with NMS as compared to C3^−/−^ MS (Fig. 3A). Microscopic imaging with the nuclear fluorophore 4′,6-diamidino-2-phenylindole (DAPI) to illuminate BMM nuclei and an anti-*Rickettsia* antibody to visualize the bacteria showed dramatically fewer *R. australis* within BMMs cultivated in NMS than in C3^−/−^ MS at 3 days post infection (Fig. 3B). Quantification of fluorescent bacilli within 600 BMMs further demonstrates the reduced *R. australis* growth resulting from complement activation (Fig. 3C).

To investigate if complement-associated restriction of *R. australis* growth in BMM was due to anaphylatoxin activities, we propagated *R. australis* in BMM with complement-deficient media (C3^−/−^ MS) supplemented with anaphylatoxin peptides. As shown in Fig. 3D, supplementation of the complement-deficient media with C3a, C5a, or C3a + C5a anaphylatoxins resulted in decreased *R. australis* proliferation at day three post infection as compared to mock-treated BMM. This is further visualized by microscopic analysis of *Rickettsia* content in BMM (Fig. 3E) and quantified as the ratio of bacilli per host nucleus in 600 individual BMMs (Fig. 3F). Together, the data presented in Fig. 3 demonstrate that complement and, more specifically, anaphylatoxin activation are sufficient to restrict *R. australis* proliferation in B57B/6J BMMs.

**FIG 4:**
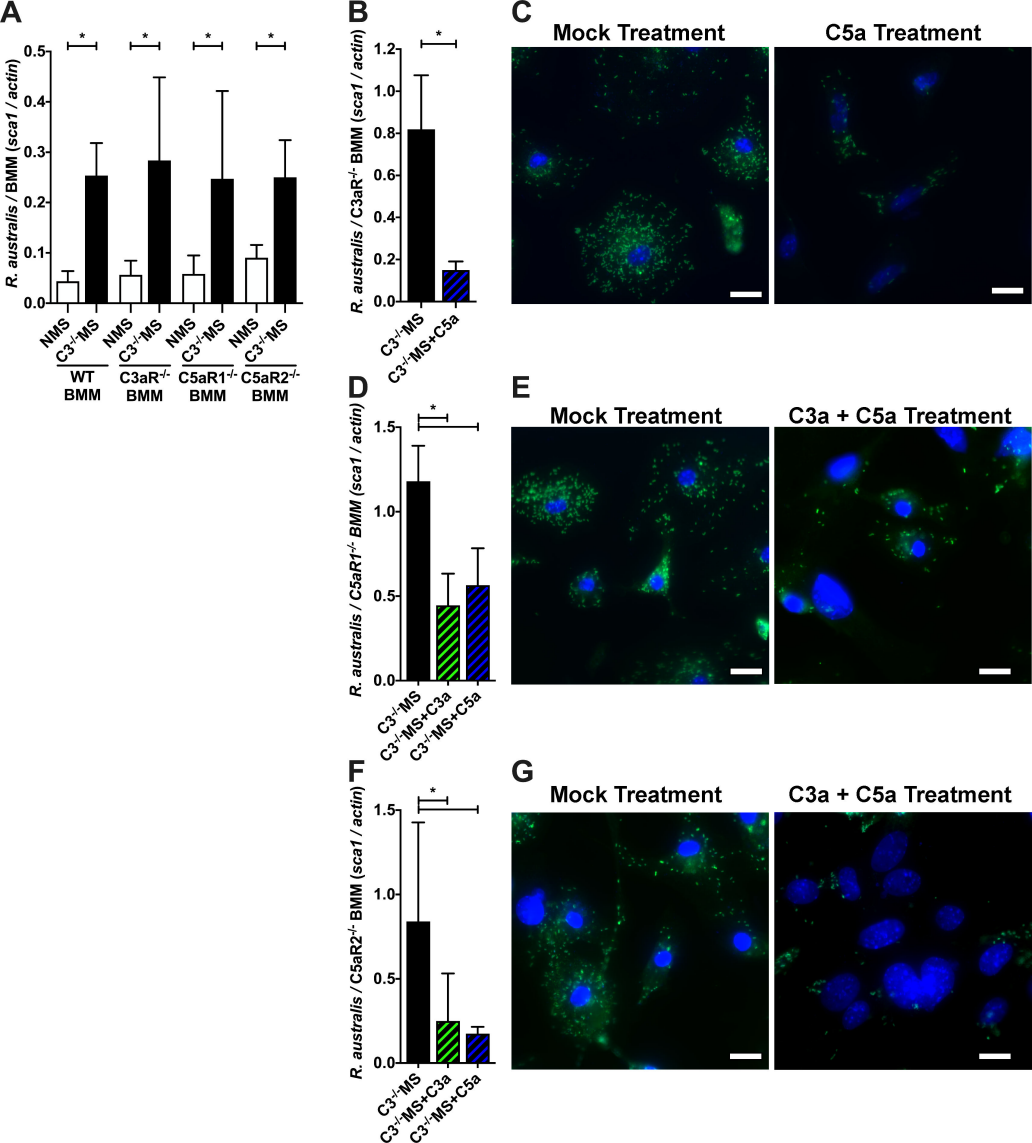
The anaphylatoxin receptors C3aR1, C5aR1, and C5aR2 are essential to the restriction of *Rickettsia australis* growth in murine BMM. (**A**) Quantity *R. australis* after 3 days of growth in WT, C3aR^−/−^, C5aR1^−/−^, and C5aR2^−/−^ BMMs cultivated in the presence of complement-active NMS or complement-deficient C3^−/−^ MS as determined by the qPCR ratio of *R. australis sca1* to murine *actin* DNA. (**B, C**) *R. australis* growth in C3aR^−/−^ BMM cultured with complement-deficient C3^−/−^ mouse serum supplemented with PBS or mouse C5a peptide as determined by qPCR (**B**) and *Rickettsia* (green) and DAPI (blue) microscopy (**C**). (**D,E**) Quantity of *R. australis* after 3 days of growth in C5aR1^−/−^ BMM cultured with complement-deficient C3^−/−^ mouse serum supplemented with PBS, mouse C3a peptide, or mouse C5a peptide as determined by qPCR (**D**) and *Rickettsia* (green) and DAPI (blue) microscopy (**E**). (**F, G**) Quantity of *R. australis* after 3 days of growth in C5aR2^−/−^ BMM cultured with complement-deficient C3^−/−^ mouse serum supplemented with PBS, mouse C3a peptide, or mouse C5a peptide as determined by qPCR (**F**) and *Rickettsia* (green) and DAPI (blue) microscopy (**G**). *P < 0.05 by (A) one-way ANOVA with Sidák’s multiple comparison of matched receptor mutants; (B,D,F) Student’s *t*-test. Scale bar = 20 µm. All columns represent at least five data points, and experiments were repeated to ensure reproducibility.

The major advantage of querying the *R. australis* + C57BL/6J system is the availability of genetically modified derivatives within that murine genetic backbone. This allows investigation of anaphylatoxin efficacy in BMMs lacking the relevant complement receptors C3aR, C5aR1, and C5aR2. To that end, we investigated the role of the different anaphylatoxin receptors in *R. australis* infected BMMs. BMMs from wild-type (WT), C3aR^−/−^, C5aR1^−/−^, and C5aR2^−/−^ mice were infected with *R. australis* in the presence of NMS or C3^−/−^ MS. As shown in Fig. 4A, BMMs cultivated in C3^−/−^ MS were more permissive for the growth of the bacteria than NMS regardless of the absence of any single anaphylatoxin receptor, demonstrating that elimination of any single receptor is not sufficient to eliminate the growth control phenotype.

By pairing individual anaphylatoxin peptides with BMMs lacking the corresponding anaphylatoxin receptor(s), we were able to interrogate each individual anaphylatoxin/anaphylatoxin receptor pair. To identify which peptide/receptor pairs are responsible for restriction of *Rickettsia* proliferation, we infected complement receptor-deficient BMMs within complement-deficient media (C3^−/−^ MS), but supplemented with either C3a or C5a peptides. As shown in Fig. 4B and C, the addition of C5a peptide to C3aR^−/−^ BMM is sufficient to restrict bacterial proliferation, demonstrating that the interaction of C5a with its corresponding receptors, C5aR1 or C5aR2, restricts *R. australis* growth. C3a was not used in this experiment because the C3aR^−/−^ cells cannot detect C3a. Additionally, supplementation of C5aR1^−/−^ BMM with either C3a or C5a peptides is sufficient to reduce *Rickettsia* growth, demonstrating that both the C3a/C3aR and C5a/C5aR2 pairs are capable of constraining bacterial proliferation (Fig. 4D and E). Finally, addition of either C3a or C5a peptides to C5aR2^−/−^ BMM can decrease *R. australis* growth, demonstrating that C3a/C3aR and C5a/C5aR1 interactions restrict bacterial growth (Fig. 4F and G). Together, these data suggest that any of C3a/C3aR, C5a/C5aR1, or C5sba/C5aR2 is sufficient to restrict *R. australis* proliferation in BMMs. Sufficiency of the C3a/C3aR and C5a/C5aR1 pairs was expected, as these are the long-standing proinflammatory anaphylatoxin/receptor pairs (54). However, the efficacy of the C5a/C5aR2 pair in restricting *Rickettsia* proliferation was unanticipated, as a consensus role for this agonist/receptor pair in mammalian cell biology is unresolved, but existing data often suggest a lack of immune activation through C5aR2-mediated signaling (67 – 69).

### Anaphylatoxin agonism modulates macrophage immune phenotypes to control *Rickettsia* proliferation

**FIG 5:**
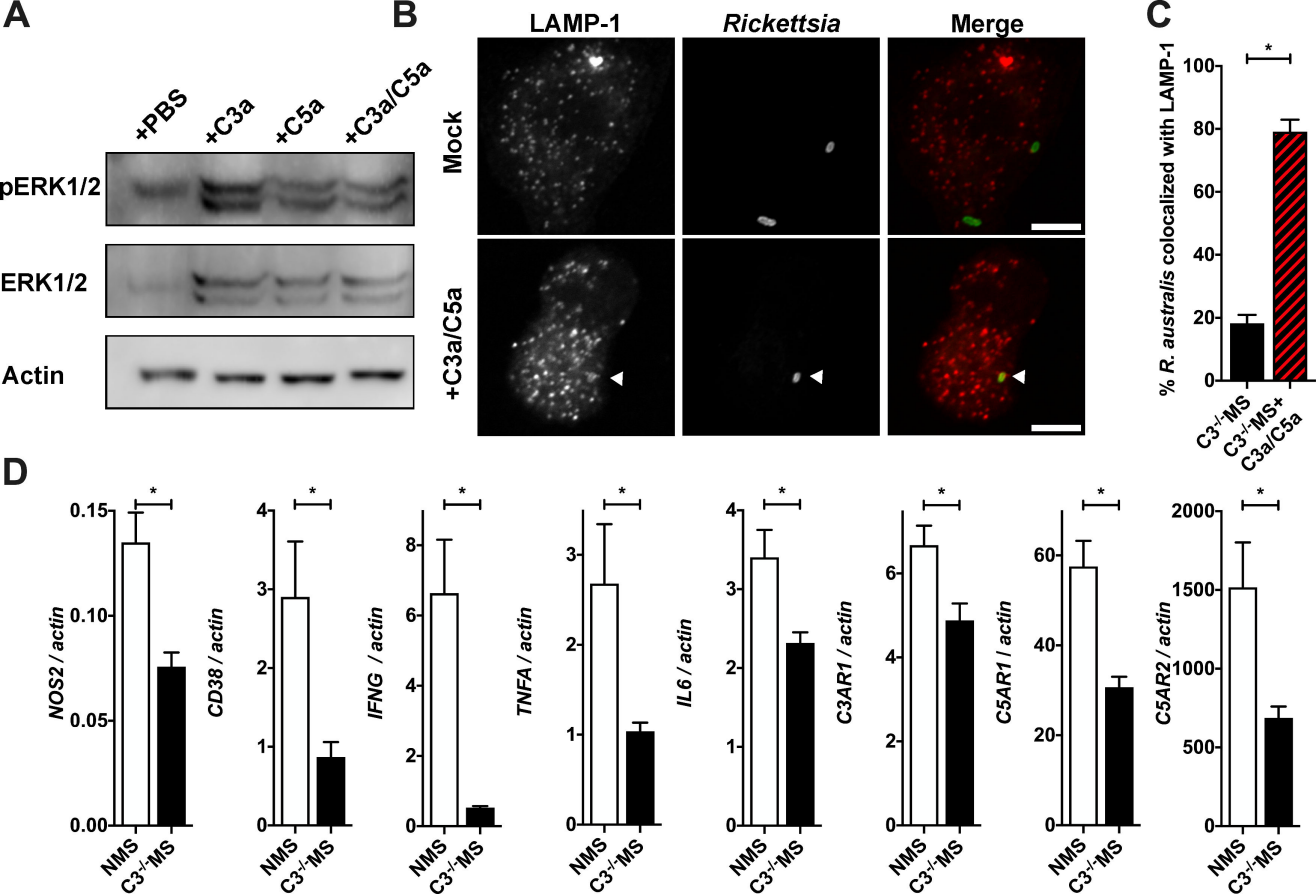
Anaphylatoxins modulate the phenotype of murine BMM, leading to decreased *Rickettsia* proliferation. (**A**) ERK1/2 phosphorylation as compared to total ERK1/2 and actin from *R. australis*-infected BMMs treated with PBS or anaphylatoxins C3a and C5a. (**B**) Representative images of *R. australis* cultured for 1 h in BMM pretreated with complement-deficient C3^−/−^ MS or C3^−/−^ MS supplemented with mouse C3a and C5a peptides as determined by confocal microscopy using the endosomal/lysosomal marker lysosomal-associated membrane protein 1 (LAMP-1), anti-*Rickettsia*, and DAPI. (**C**) Subsequent quantification of *R. australis*-LAMP1 co-localization in over 100 single BMMs. (**D**) Quantification of cDNA from *Rickettsia*-infected BMMs cultured in the presence of complement-active NMS or complement-deficient C3^−/−^ MS. Macrophage activation markers, pro-inflammatory cytokines, and anaphylatoxin receptor mRNA were quantified by qPCR as compared to *actin* cDNA. *P < 0.05 by Students *t*-test. Scale bar = 5 µm. All columns represent at least five data points, and experiments were repeated to ensure reproducibility. LAMP-1 colocalization was observed for 100 individual cells in each column and was repeated to ensure reproducibility.

Having established a role for anaphylatoxin/anaphylatoxin receptors in restricting the proliferation of multiple *Rickettsia* species within multiple different host cell types, we sought to identify the physiological changes occurring within infected monocytes that ultimately block *Rickettsia* proliferation. A central cell signaling phenotype associated with anaphylatoxin agonism is phosphorylation of ERK1 and ERK2 protein-serine/threonine kinases (42, 70 – 73). To investigate if exogenous anaphylatoxin stimulation also induced ERK1/2 phosphorylation in *R. australis*-infected BMMs, the host cells were infected with *R. australis* and treated with anaphylatoxin peptides for a total of 10 min. As shown in Fig. 5A, *R. australis*-infected BMMs treated with anaphylatoxins demonstrate greater ERK1/2 phosphorylation after 10 min of infection than mock-treated *R. australis*-infected BMMs, suggesting that anaphylatoxin agonism of anaphylatoxin receptors induces rapid signal transduction resulting in the phosphorylation of ERK1/2.

With the knowledge that some innate immune cells are capable of preventing the essential rickettsial pathogenic act of phagosomal escape, we subsequently queried for anaphylatoxin-induced changes to *Rickettsia* localization within host cells (5, 66, 74). C57BL/6J BMMs cultured in complement-deficient serum were treated with either PBS or C3a + C5a anaphylatoxins. After 10 min of anaphylatoxin stimulation, the BMM were infected with *R. australis*. After an additional 1 h of incubation, the samples were fixed with paraformaldehyde to halt all progression through the invasion process. Phagosomal escape was assessed by confocal microscopy using lysosomal-associated membrane protein 1 (LAMP-1) and anti-*Rickettsia* antibodies. As shown in Fig. 5B, more bacilli co-localize with LAMP-1 in anaphylatoxin-treated BMMs than their mock-treated counterparts. Quantifying the frequency of colocalization indicates that anaphylatoxin treatment results in a significant increase in LAMP-1-positive *Rickettsia* (Fig. 5C), suggesting that anaphylatoxin signaling induces BMMs to block or delay *R. australis* phagosomal escape.

Finally, anaphylatoxin/anaphylatoxin receptor activation is known to induce global changes to the transcriptional profile of leukocytes (75, 76). Previous studies have shown that secretion of cytokines, including IL-6, TNF-α, and IFN-γ, is associated with inhibition of *Rickettsia* growth (48, 77 – 82). To determine if anaphylatoxin treatment changes the transcription of inflammation-associated transcripts within infected cells, we again infected WT BMMs with *Rickettsia* in the presence of complement-active NMS or complement-deficient C3^−/−^ MS. Infected BMMs were harvested 24 h post infection to assess the quantity of mammalian transcripts by qPCR. The quantity of each transcript was equilibrated by comparison to actin mRNA. As shown in Fig. 5D, the quantity of transcripts encoding for the cytokine inducible nitric oxide synthase iNOS *(NOS2*), the M1 macrophage marker CD38 (*CD38*), and the cytokines IFN-γ (*IFNG*), TNF-α (*TNFa*), and IL-6 (*IL6*) were all increased with complement activation, indicating that anaphylatoxin agonism induces transcriptional reprogramming within the infected BMMs. mRNA encoding for the anaphylatoxin receptors was also increased, illuminating a potential feedback loop. Together, the observed increase in (i) ERK1/2 phosphorylation, (ii) localization of *Rickettsia* in phagosomes, and (iii) levels of mRNA encoding for pro-inflammatory markers indicates that anaphylatoxin stimulation activates macrophages to control the proliferation of pathogenic *Rickettsia*.

## DISCUSSION

Herein, we report a role for anaphylatoxins and anaphylatoxin receptors in reducing intracellular growth of pathogenic *Rickettsia* in monocytic host cells. Stimulation with either C3a or C5a is sufficient to induce murine RAW264.7, human THP-1, and murine bone marrow macrophages to directly control the proliferation of three different rickettsial pathogens, including *R. parkeri*, *R. rickettsii*, and *R. australis*. Additionally, anaphylatoxin stimulation of THP-1 macrophages impacts *Rickettsia* survival in endothelial/macrophage co-cultures. The growth restriction phenotype is induced by the presence of both anaphylatoxin and cognate receptors, with C3a/C3aR, C5a/C5aR1, and C5a/C5aR2 pairs each contributing to efficacy. Finally, anaphylatoxin stimulation of *Rickettsia-*infected bone marrow macrophages produces proinflammatory phenotypes that correlate with a reduction in intracellular growth. Together, these data demonstrate that *Rickettsia*-induced anaphylatoxin production acts upon leukocytes to control intracellular *Rickettsia* replication.

When viewing the host-pathogen interaction through the lens of the host cell, we currently understand that macrophages have the capacity to mount an innate immune response when infected by *Rickettsia* pathogens (83). In this manuscript, we have established that activation of the anaphylatoxin-anaphylatoxin receptor axis modifies monocytic cells to control *Rickettsia* infection. The individual steps that connect anaphylatoxins to restricted *Rickettsia* proliferation include the following: (i) the presence of extracellular anaphylatoxins C3a or C5a; (ii) recognition of these peptides by the cognate anaphylatoxin receptors C3aR, C5aR1, or C5aR2; (iii) induction of intracytoplasmic cell signaling involving ERK1/2 phosphorylation; and (iv) changes to the physiology of the mammalian cell that include restriction of rickettsial phagosomal escape and differential transcriptional programming.

The requirement for extracellular anaphylatoxins correlates with other exogenous signals that can activate both infected leukocytes and endothelial cells to control *Rickettsia* proliferation. The chemokines CCL5, CXCL5, and CXCL10 and the cytokines IFN-γ, TNF-α, IL-1α, IL-1β, IL-6, IL-8, IL-12, and IL-18 have all been associated with activation of *Rickettsia*-infected endothelial cells or leukocytes (16, 17, 84 – 88). The primary difference that separates anaphylatoxins from these previously identified classical immunostimulants is that chemokines and cytokines are largely produced *de novo* by cells after detection of a pathogen, whereas anaphylatoxins are produced by proteolysis of preformed precursors independent of a host cell. Due to the speed of anaphylatoxin production, it is reasonable to postulate that anaphylatoxin stimulation precedes the activation of innate immunity in earnest.

Extracellular C3a and C5a peptides are detected by the cell surface anaphylatoxin receptors C3aR, C5aR1, and C5aR2 (Fig. 1G; Fig. 2D; Fig. 4; Fig. 5A). Additionally, each individual peptide/receptor pair was sufficient to mediate restriction of *Rickettsia* growth and ERK1/2 phosphorylation, with no observed additive effect with the application of both anaphylatoxins (Fig. 1E and F; Fig. 2C; Fig. 3D). Previous studies have observed a synergetic effect when endothelial cells are treated simultaneously with C3a and C5a to release IL8 (89), but not IL-6 or IFN-γ and IL-2 responses in the whole blood of healthy donors after stimulation by hepatitis B virus antigens (90). Although we never observed a differential or synergistic effect of different anaphylatoxins, it may remain worthwhile to investigate how each anaphylatoxin integrates into our understanding of the immune response to *Rickettsia* infection. The anaphylatoxin receptors therefore join an ever-increasing repertoire of innate immune receptors that can indirectly or directly sense the presence of *Rickettsia* bacilli. Previous findings have demonstrated that the innate immune system can directly detect *Rickettsia* through preexisting IgM molecules that activate the complement system to produce anaphylatoxins (91). Therefore, the pathway from (i) IgM recognition of the bacterial surface, (ii) anaphylatoxin production, and (iii) anaphylatoxin receptor agonism is a strong surveillance pathway for alerting mammalian cells about the presence of *Rickettsia*. Anaphylatoxin-mediated immunosurveillance joins other previously identified mechanisms that include Toll-like receptor 4 and a cytoplasmic sensor connected to the NLRP3 inflammasome (83, 92 – 95). Our current knowledge posits that these three surveillance mechanisms are the initiators of the innate immune response to *Rickettsia* infection.

In Fig. 5A, we have established that anaphylatoxin receptor activation induces cell signaling changes within the infected cell that include phosphorylation of ERK1 and ERK2. ERK1/2 signaling has previously been connected to other related Rickettsiales. *Anaplasma phagocytophilum* and *Orientia tsutsugamushi* both induce ERK1/2 phosphorylation, but *Ehrlichia chaffeensis* infection of macrophages decreases ERK1/2 phosphorylation (96 – 101). As such, anaphylatoxin-induced ERK1/2 activation is correlative to the “activated” cell signaling profile that supports host control of *Rickettsia* growth (102). Many different cell signaling proteins, including MyD88, NF-κB, ISG15, SOCS1/UBP43, and p38, have been identified as correlates of productively activated monocytic or endothelial cells (103 – 107). The relative importance of each of these host activation markers has yet to be determined, but together, these proteins serve as the foundation for examining how *Rickettsia* “deactivates” infected host cells. Rickettsial manipulation of host signaling is currently exemplified at a molecular level by modulation of PI3K and Arf6 activity but is also apparent by noninflammatory changes to transcriptional and proteomic profiles induced by infection (15, 60, 108 – 110). It is clear that there is an ongoing battle for cell signaling mechanisms occurring within the cytoplasm of *Rickettsia*-infected leukocytes.

The final outcome of anaphylatoxin stimulation is the induction of changes to the physiology of the host monocyte. At the most basic level, the conflict between *Rickettsia* and a host leukocyte is summarized as bacterial proliferation within the host cytoplasm versus intracellular killing of the bacteria through (i) inducible nitric oxide synthesis, (ii) hydrogen peroxide and reactive oxygen species production, and (iii) sequestration of tryptophan (48, 111, 112). The time it takes to fully implement these antibacterial strategies may explain why we only observed a restriction of *Rickettsia* growth after 3 days of infection. Our observed anaphylatoxin-induced increase in *Rickettsia*-LAMP1 colocalization (Fig. 5B and C), induction of iNOS transcription (Fig. 5D), and induction of the macrophage activation marker CD38 (Fig. 5D) strongly supports the concept that anaphylatoxin stimulation leads to intracellular killing of *Rickettsia* (113 – 116). This model implies that the cell that detects anaphylatoxins is the same cell that induces bactericidal mechanisms. As demonstrated by the changes to the transcription of multiple proinflammatory genes (Fig. 5D) and activated-monocyte restriction of rickettsial proliferation in endothelial cells (Fig. 2F), anaphylatoxin stimulation also has indirect effects on the larger population. Indeed, increased transcription of genes encoding for the cytokines IFN-γ, IL-6, and TNF-α all support the concept that anaphylatoxin production is a comprehensive activator of innate immunity beyond the specific monocyte that detects the presence of C3a or C5a (16, 117 – 119). Our data show that complement activation and anaphylatoxins drive *Rickettsia*-infected macrophages toward an inflammatory phenotype through the modulation of cytokine transcription.

The complement components C3a/C5a and the cognate anaphylatoxin receptors C3aR and C5aR1 have well-defined and broad roles in inflammation and induction of the adaptive immune response to infection (120, 121). As complement is largely an immunosurveillance network, most studies have investigated the link between anaphylatoxins and innate immune cells. These studies have identified anaphylatoxin-induced activation of macrophages, eosinophils, basophils, neutrophils, and mast cells (54, 122 – 126), whereby unregulated anaphylatoxin production or reduced degradation is linked to immunopathologic conditions, including autoimmunity, toxic shock, chronic inflammation, and excessive inflammation during severe severe acute respiratory syndrome coronavirus 2 infection (127 – 132). Conversely, genetic or chemical inhibition of anaphylatoxin receptor activity decreases immune clearance of the extracellular pathogens *Pseudomonas aeruginosa*, *Streptococcus* spp., *Staphylococcus* spp., and *Neisseria meningitidis* (133 – 141). Importantly, anaphylatoxin activity has also been connected to the control of intracellular bacterial pathogens, including *Mycobacterium bovis*, *Listeria monocytogenes*, and *Chlamydia psittaci* (142 – 145). With *Rickettsia* joining the ranks of anaphylatoxin-sensitive intracellular pathogens, there appears to be a growing consensus that anaphylatoxin receptor agonism augments the ability of leukocytes to control intracellular bacterial infections.

Activation of endothelial cell signaling by *Rickettsia* infection induces the production of cytokines and the recruitment of many different innate and adaptive immune cell types (13, 117). As shown in Fig. 2E and F, EA.hy926 is unable to restrict *Rickettsia* proliferation after anaphylatoxin stimulation. However, the addition of anaphylatoxin-activated macrophages is sufficient to reduce *Rickettsia* proliferation in the entire culture. While the mechanism of monocyte/endothelium communication is not elucidated in the present study, previous studies have highlighted that co-culture of macrophages modified the transcriptional profile of endothelial cells (146). Additionally, infection of endothelial cells with *Rickettsia* attracts leukocytes to adhere to the infected endothelial cell, presumably to help resolve the local infection (147, 148). As such, there is a clear two-way communication between endothelial cells and monocytes, whereby infected endothelial cells recruit leukocytes, which in turn activate the bactericidal mechanisms within the infected endothelial cells (149 – 152). As the anaphylatoxin/anaphylatoxin receptor axis enhances macrophage-dependent restriction of *Rickettsia* growth within endothelial cells, anaphylatoxin production is likely to be one of these chemical messengers responsible for endothelium/macrophage communication.

Two reports have linked anaphylatoxin receptor overexpression to disruption of macrophage functionality (153, 154). As shown in Fig. 5C, anaphylatoxin stimulation increased transcription of the three anaphylatoxin receptors, C3aR, C5aR1, and C5aR2. Therefore, in the C57BL/6J BMM model system, anaphylatoxin signaling induces a feedback loop that increases expression of the anaphylatoxin receptors. Anaphylatoxin-stimulated monocytes may elaborate increased sensitivity to further anaphylatoxin stimulation, raising the specter of immunopathogenesis associated with overabundant anaphylatoxin/anaphylatoxin receptor axes.

The data presented in this manuscript integrates into multiple ongoing investigations into the *Rickettsia-*monocyte interaction. These studies are largely derived from the concept that endothelial cells are “susceptible” to *Rickettsia* parasitism, but activated leukocytes have the ability to both directly combat infection and activate nearby cells to generate an overall environment less hospitable to *Rickettsia* infection. Supported by the observation of pathogenic *Rickettsia* within monocytic cells *in vivo* and *Rickettsia akari* tropism for monocytes (28, 155, 156), the *Rickettsia-*monocyte host-pathogen interaction has developed into a significant line of inquiry (5, 47, 59). From the pathogen perspective, some *Rickettsia* have evolved mechanisms to survive and replicate in macrophages. Indeed, proliferation within cultured macrophages is a hallmark of pathogenic species that is lacking in the nonpathogenic species (15, 59). *Rickettsia* infection of THP-1 macrophages leads to the activation of the lipid catabolic pathway and induces reprograming of the cells toward an anti-inflammatory profile (15, 157). Furthermore, *R. australis* can establish a cytosolic niche in macrophages by inhibiting the bactericidal effect mediated by IL-1β via the Atg5-dependent autophagy response, leading to systemic infection of *R. australis* (158, 159). Finally, *R. parkeri* OmpB blocks polyubiquitylation to promote autophagy evasion in macrophages and is shown to be essential for bacterial growth (160). Together, these data demonstrate that *Rickettsia* species have evolved molecular mechanisms to deactivate monocytic cells. However, when examining the complex interplay ongoing within *Rickettsia*-infected monocytes, anaphylatoxin signaling lies well within the host repertoire of antibacterial mechanisms.

In summary, we have utilized models of *Rickettsia* infection in monocytes to elucidate the role of complement activation and the anaphylatoxin/anaphylatoxin receptor axis in modulating the response to infection. Anaphylatoxins were found to activate macrophages to restrict *Rickettsia* infection by limiting phagosome escape. Activated macrophages accumulate pERK1/2 and increase transcription of monocyte activation markers and pro-inflammatory cytokines. Overall, our work illuminates the role of the anaphylatoxin/anaphylatoxin receptor axis in the innate immune control of *Rickettsia* parasitism.

## MATERIALS AND METHODS

### Murine, cell, and *Rickettsia* lines

C57BL/6J (WT); complement component C3^−/−^ (B6;129S4-C3^tm1Crr^/J) (46); complement receptor C3aR^−/−^ [B6.129S4(C)-C3ar1^tm1Cge^/Baoluj] (161); complement receptor C5aR1^−/−^ [B6.129S4(C)-*C5ar1^tm1Cge^*/BaoluJ] (162); and complement receptor C5aR2^−/−^ mice (B6.129S1-*C5ar2^tm1Cge^*/HgrJ) (163) mice were used in this study. WT animals were acquired from Jackson Laboratory and were age- and sex-matched to the internally bred genetically modified animals.

All mammalian cells were cultured at 37°C with 5% CO_2_. EA.hy296 (164), RAW264.7, and Vero cells were routinely grown in complete DMEM (cDMEM) consisting of Dulbecco’s modified Eagle’s medium (Gibco) supplemented with 10% heat-inactivated fetal bovine serum (FBS; Atlanta Biologicals), HyClone MEM nonessential amino acid solution (Thermofisher), and 1× L-glutamine (Gibco). THP-1 cells were routinely grown in complete RPMI (cRPMI) consisting of RPMI 1640 media (Gibco) supplemented with 10% heat-inactivated fetal bovine serum, HyClone MEM nonessential amino acid solution, and 1× L-glutamine. Media from routine culture were removed and replaced where noted.

*R. parkeri* Portsmouth, *R. australis* Cutlack, and *R. rickettsii* Sheila Smith were cultivated in Vero cells, lysed using blunt cannulas, and sucrose-gradient purified as previously described (165). Rickettsial stocks were aliquoted in sucrose-phosphate-glutamate buffer and stored at −80°C until use. The quantity of bacteria was determined by titration, as has been previously described (166).

### Serum and tissues

Pooled NHS was purchased from Innovative Research. NHS was heat-inactivated at 56°C for 30 min or with 20 mM EDTA where noted (62, 167). NMS (C57BL/6J) and C3^−/−^ MS were isolated by cardiac puncture and Z-gel (Sarstedt) centrifugation. All serum samples were aliquoted, snap-frozen, stored at −80°C, and thawed immediately before use.

To generate BMMs, tibial and humeral marrow was extracted from 8- to 12-week-old male WT, C3aR^−/−^, C5aR1^−/−_,_^ and C5aR2^−/−^ mice (168 – 170). The cells were centrifuged and resuspended in DMEM medium supplemented with 20% FBS, 5% HyClone MEM nonessential amino acid solution, 1× L-glutamine, 50 μg/mL carbenicillin, and 10 ng/mL macrophage colony-stimulating factor (Thermofisher). The isolated bone marrow cells were cultured at 37°C with 5% CO_2_ for 5 days with media replacement on day 3. Then, 24 h prior to infection, the cells were liberated with StemPro Accutase Cell Dissociation Reagent (Thermofisher), washed to remove antibiotics, and plated at 5 × 10^4^ cells/well in media lacking M-CSF and carbenicillin. All infection assays were performed using 6–9-day-old BMMs.

### *Rickettsia* infections

RAW264.7 cells were cultured in 96-well plates for 24 h in cDMEM. The media were removed from cells and replaced with DMEM media with 10% NMS or C3^−/−^ MS. After 20 min, cells were infected with *Rickettsia* spp. at multiplicity of infection (MOI) = 1. THP-1 and EA.hy926 cells were seeded in 96-well plates for 24 h in cRPMI or cDMEM. The media were removed from cells and replaced with media with either 10% normal human serum, heat-inactivated human serum, or EDTA-inactivated human serum. After 20 min, the cells were infected by *Rickettsia* species at MOI = 1.

For EA.hy926 + THP-1 co-culture, 1 × 10^4^ EA.hy926 cells were seeded in 96-well plates with cDMEM media. Then, 15 h later, cells were infected with MOI = 1 *R*. *parkeri*. After 24 h, the media were replaced with (i) complete media, (ii) 1 × 10^4^ THP-1 in complete media, or (iii) 1 × 10^4^ THP-1 in RPMI media supplemented with 10% NHS. The cells were then cultivated for an additional 3 days before analysis.

For BMM infections, the M-CSF-induced BMMs were seeded in a 96-well plate at 5 × 10^4^ cells/well. Prior to infection, the media were removed and replaced with DMEM media containing NMS or C3^−/−^ mouse serum in the presence or absence of anaphylatoxin. The BMMs were infected with *R. australis* at MOI = 1 and incubated for an additional 3 days before analysis.

### Anaphylatoxin treatments

To analyze anaphylatoxins, RAW264.7 and BMMs were cultured in DMEM media supplemented with 10% C3^−/−^ mouse serum. Cells were treated with PBS (mock) or the synthetic mouse anaphylatoxins 1 µg/mL C3a, 1 µg/mL C5a, or 1 µg/mL C3a + 1 µg/mL C5a (RD Systems). Cells were incubated for 20 min before infection by *Rickettsia* at MOI = 1 and were then incubated for 3 days before analysis. To analyze human anaphylatoxins, THP-1 cells were cultured in RPMI media supplemented with 10% heat-inactivated human sera. Cells were treated with PBS or synthetic human anaphylatoxins 1 µg/mL C3a, 1 µg/mL human C5a, or 1 µg/mL C3a and C5a (RD Systems). After incubation for 20 min, the THP-1 cells were infected with *Rickettsia* at MOI = 1 and incubated for 3 days before analysis.

### Anaphylatoxin receptor antagonists

Media were supplemented with the C3a receptor antagonist 60 nM SB290170 (Sigma) or C5a receptor antagonist 40 nM PMX53 (Sigma) as described (56, 57). Cells were pretreated for 20 min before infection by *Rickettsia* at MOI = 1 and were incubated for an additional 3 days before DNA extraction.

### Assessment of cell viability

At the time points indicated for each cognate qPCR, a CyQUANT MTT Cell Viability Assay (Thermofisher) was performed on the live cells following the manufacturer’s recommendation. The absorbance was read at 540 nm using a Spectramax M2 microplate reader (Molecular Devices), and the data are graphed as the absorption of at least four different samples.

### Immune correlates of activation

C3a and C5a concentrations were determined by an enzyme-linked immunosorbent assay as previously described (91). Appropriate media were harvested on day 3 and used for the evaluation of complement activation.

To assess ERK1/2 activation, standard media were removed from murine BMMs and replaced with media containing 10% C3^−/−^ MS supplemented with either PBS or 1 µg/mL mouse anaphylatoxins C3a and C5a. These BMMs were immediately infected with *R. australis* at MOI = 1, centrifuged at 300 × *g* for 5 min, and incubated for an additional 5 min at 37°C. Cells were harvested in RIPA buffer (Thermofisher) supplemented with protease inhibitors (Pierce) and a HALT phosphatase inhibitor cocktail (Thermo). Total protein was quantified using the BCA protein assay kit (Pierce), resolved by sodium dodecyl sulfate–polyacrylamide gel electrophoresis (SDS-PAGE), and transferred to the polyvinylidene membrane (Immobilion). ERK1/2 and phosphorylated ERK1/2 were detected with rabbit anti-mouse ERK1/2 and phospho-ERK1/2 (RD Systems) and goat anti-rabbit-IgG-HRP (Invitrogen) secondary antibodies. Actin was detected by mouse anti-beta actin (Novusbio) with a goat anti-mouse IgG-HRP (Invitrogen) secondary antibody. Streptavidin-HRP (R&D systems) and crescendo western HRP substrate (Immobilon) were used before acquisition with Chemi-Imager (Azure Biosystems).

### Analysis of bacterial loads and expression of murine genes

Total genomic DNA (mouse and *Rickettsia*) was isolated using the PureLink Genomic DNA Mini Kit (Thermofisher). DNA was quantified by PCR utilizing GreenLink No-ROX QPCR Mix (BioLink) and a CFX-Connect Real-Time System (BioRad) using mouse actin (24) and human *GADPH* (171) primers that were previously described.

RNA was isolated using the RNA Mini Kit (Invitrogen), DNA was removed using the TURBO DNA-free Kit (Thermo Scientific), and cDNA was generated using the RevertAid RT Reverse Transcription Kit (Thermo Scientific). Primers used for the quantification of mouse *IFNG*, *IL6*, *NOS2*, *TNFa*, *CD38*, *C3aR*, *C5aR1*, and *C5aR2* are listed in Table S1. All samples were graphed against a standard curve of the specific amplicon cloned into the pCR2.1 plasmid. The data are expressed as the ratio of the targeted gene to *Mus musculus* actin cDNA.

### Confocal microscopy

Cells were fixed with 4% paraformaldehyde, permeabilized with 0.1% Triton X-100, and blocked in PBS containing 2% bovine serum albumin (BSA). *Rickettsia* species were detected with rabbit anti-*Rickettsia* (RcPFA) (172), followed by goat anti-rabbit Alexa Fluor 488 antibody (Invitrogen). Mammalian nuclei were identified with DAPI. LAMP-1 was stained with mouse anti-CD107a clone 1D4B (Bioscience) and followed by goat anti-mouse Alexa Fluor 546 (Invitrogen). To image total *Rickettsia*, images were captured using a Zeiss LSM 800 Laser Scanning Confocal Microscope system at 40×. *Rickettsia*/LAMP1 co-localization was assessed by consistently obtaining five slices of optical sectioning of cells using a Z step using two channels simultaneously with 63× oil immersion. Acquired images were processed on ImageJ software.

### Statistical analyses

Statistical analysis was performed on Excel and GraphPad PRISM software (version 8.3). Data from Fig. 1A, B, Fig. 2B, D, and E, Fig. 3C, F, Fig. 4B, and Fig. 5C and D were analyzed by the Student’s *t*-test. Data from Fig. 1C, D, Fig. 2A, and Fig. 3A were analyzed by a one-way ANOVA with Sidák’s multiple comparison of matched days. Data from Fig. 1E, F, and G, Fig. 2C, Fig. 3D, and Fig. 4D and F were analyzed by a one-way ANOVA with Dunnett’s multiple comparison test to mock-treated control. Data from Fig. 2F were analyzed with a one-way ANOVA with Tukey’s multiple comparison test of all data sets. Data from Fig. 4A were analyzed with a one-way ANOVA with Dunnett’s multiple comparison of each NMS + C3^−/−^ MS pair.

## SUPPLEMENTAL MATERIAL

Supplemental file 1 spectrum.02538-23-s0001.docx Fig. S1 to S5 and Table S1.

ASM does not own the copyrights to Supplemental Material that may be linked to, or accessed through, an article. The authors have granted ASM a non-exclusive, world-wide license to publish the Supplemental Material files. Please contact the corresponding author directly for reuse.
