# Supplementary material for: Anaphylatoxin signaling activates macrophages to control intracellular Rickettsia proliferation
Source: Microbiol Spectr. 2023 Oct 19;11(6):e02538-23. doi: 10.1128/spectrum.02538-23 (PMC10714731; doi:10.1128/spectrum.02538-23)
Supplement: Supplemental file 1 — Fig. S1 to S5 and Table S1. [file spectrum.02538-23-s0001.docx]

**
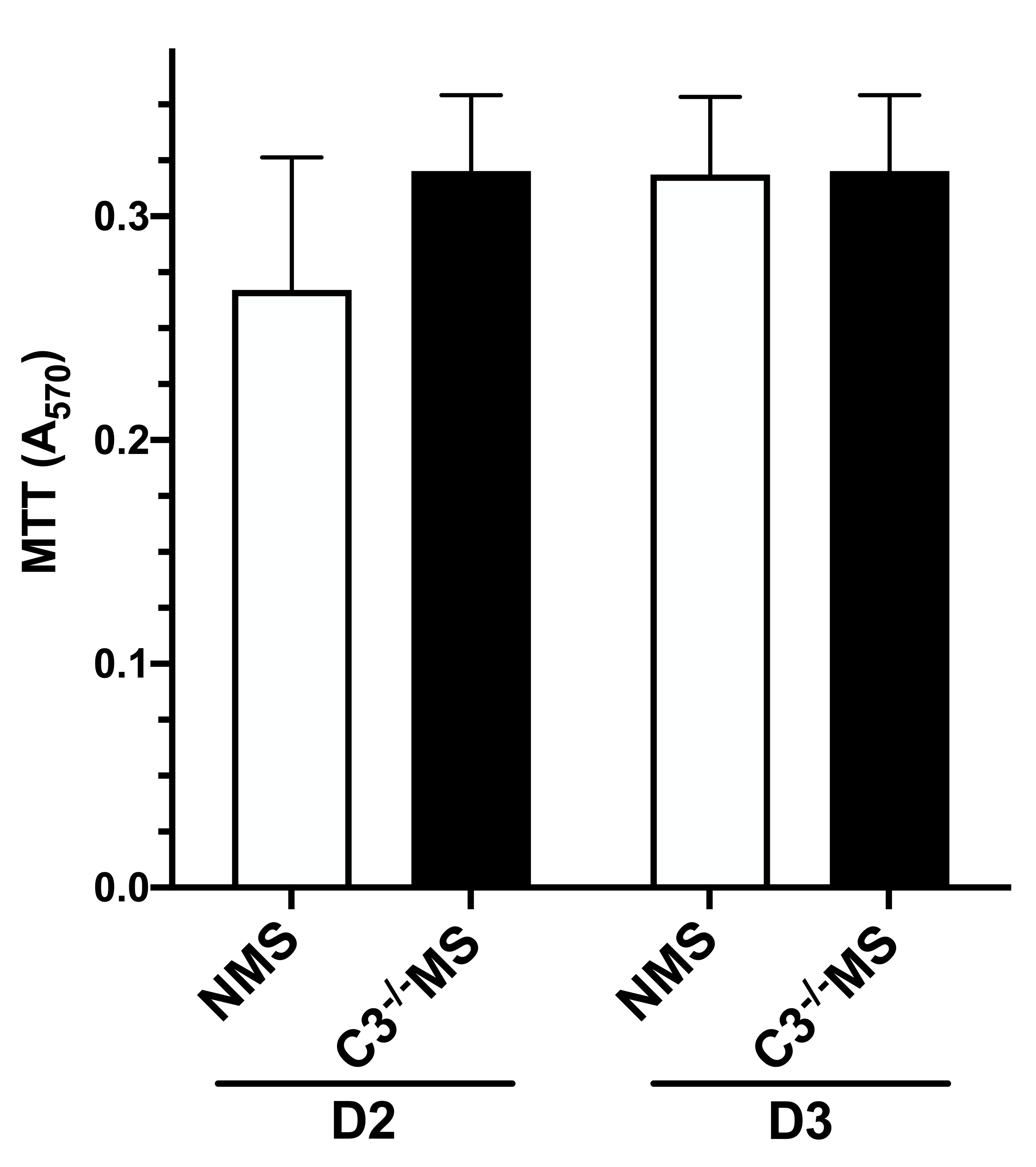
Fig S1:** Cell viability of *R. parkeri* infected RAW264.7 cells cultivated for three days with complement active serum (NMS) or complement deficient serum (C3^-/-^MS) as determined by MTT assay. No significant differences as measured by 1-way ANOVA with Sidak ‘s multiple comparison of matched days.

**
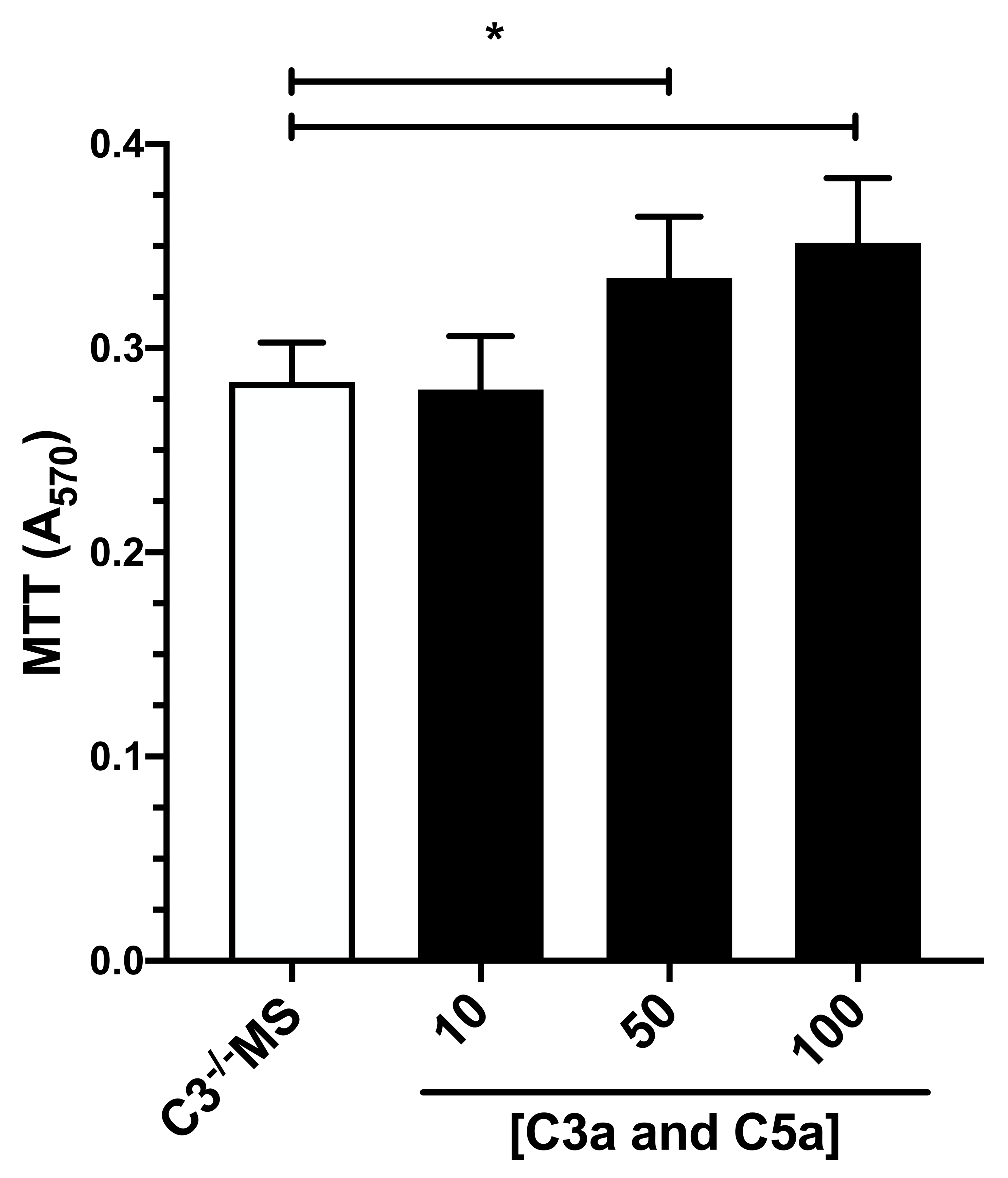
Fig S2:** Cell viability of uninfected RAW264.7 cells cultivated for 3 days with C3^-/-^MS supplemented with increasing concentrations of both C3a and C5a anaphylatoxins as determined by MTT assay. * p<0.05 increase in cell viability as measured by 1-way ANOVA with Dunnett’s multiple comparison to no anaphylatoxin (C3^-/-^MS) control.

**Fig S3:** Cell viability of uninfected or *R. parkeri* infected THP-1 cells cultivated for 3 days with normal human serum (NHS) or heat inactivated human serum (hiHS). No significant differences in cell viability as measured by 1-way ANOVA with Dunnett’s multiple comparison to uninfected NHS control.

**
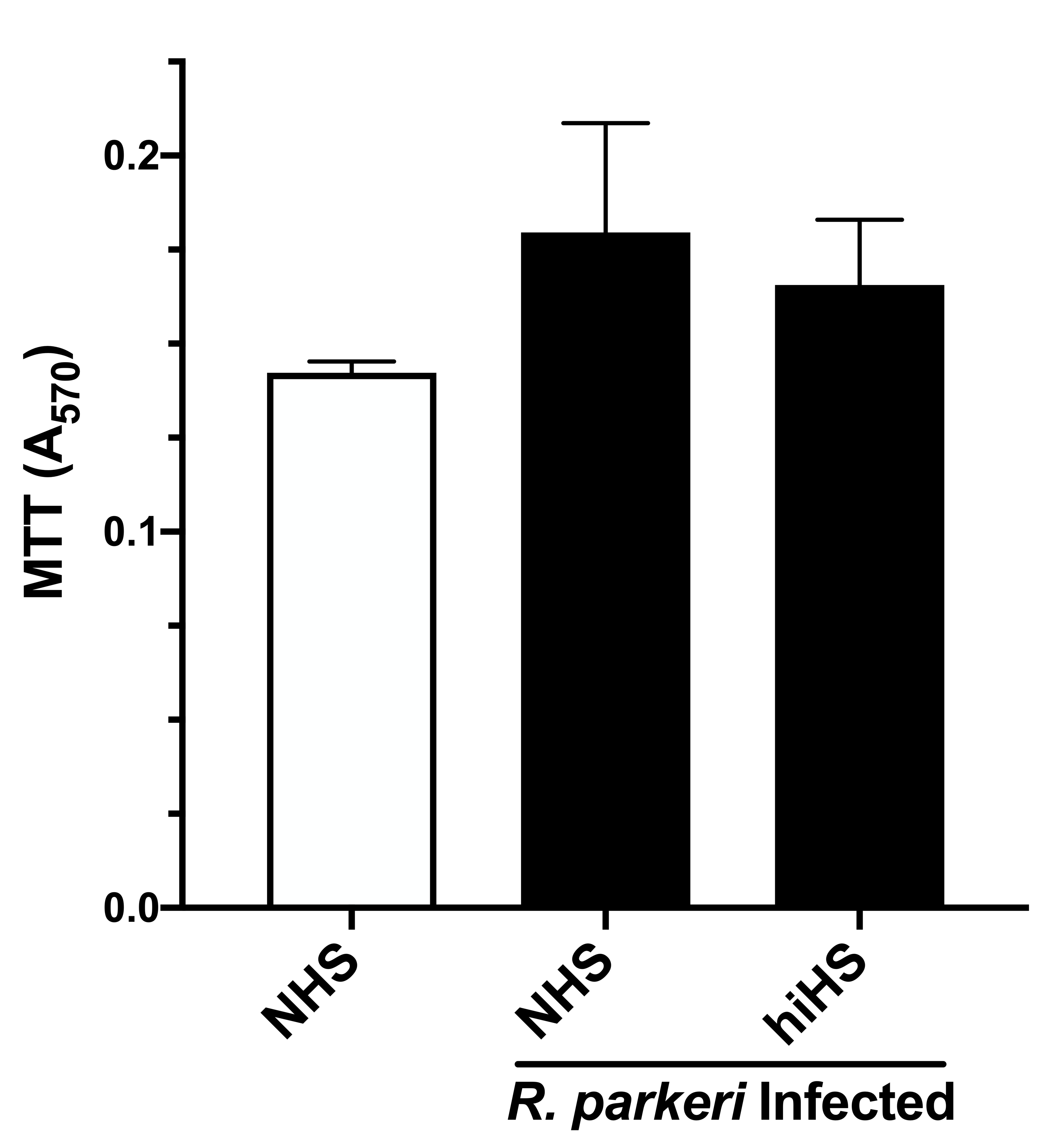
**

**Fig S4:** Cell viability of uninfected THP-1 cells cultivated for 3 days with normal human serum (NHS) or NHS supplemented with EDTA No significant differences in cell viability as measured Student’s
*t-*test.


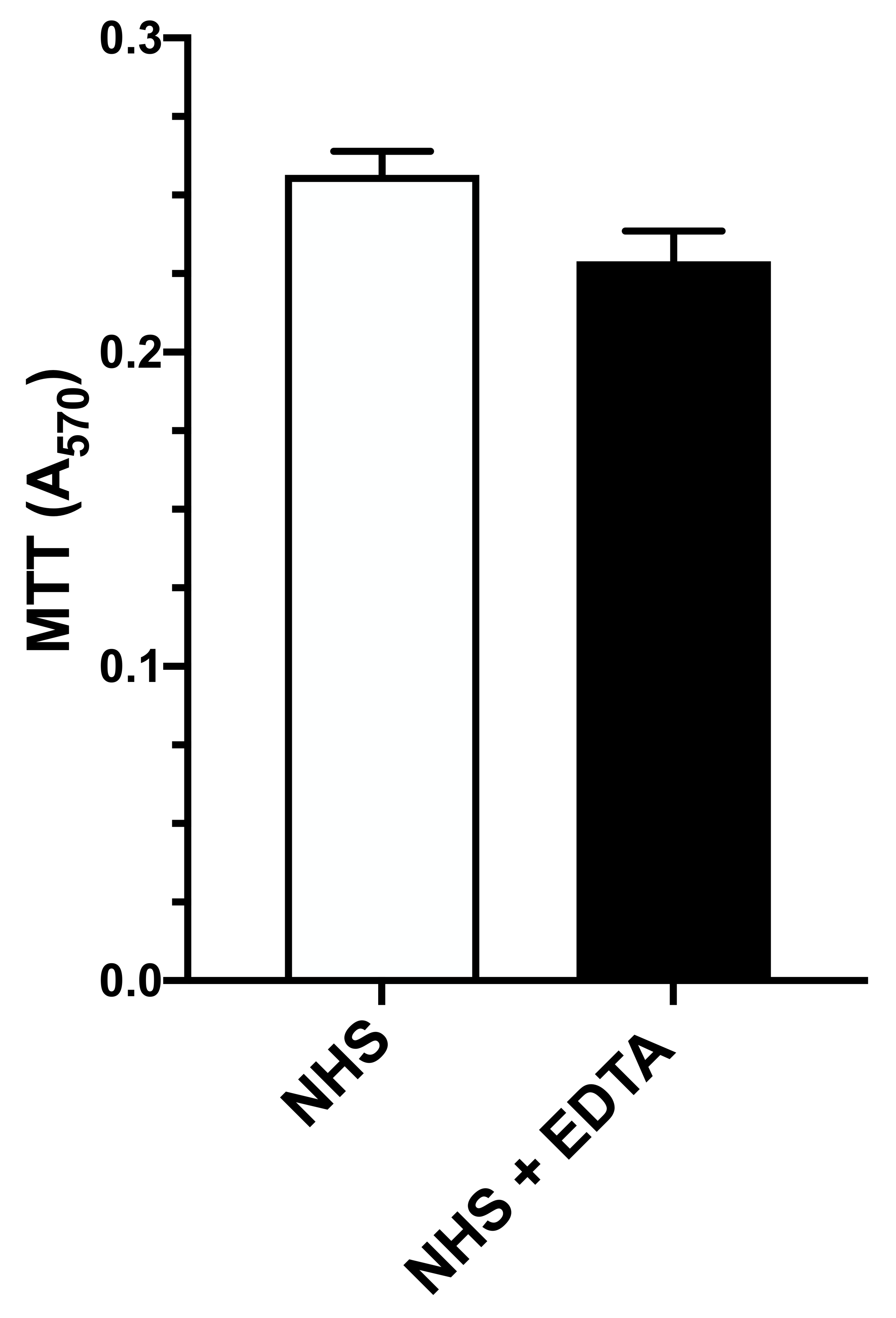


**Fig S5:** Cell viability of uninfected THP-1 cells cultivated for 3 days with described concentrations of **(A)** SB290170 or **(B)** PMX53. No significant differences in cell viability as measured as measured by 1-way ANOVA with Dunnett’s multiple comparison to each NHS control.

**
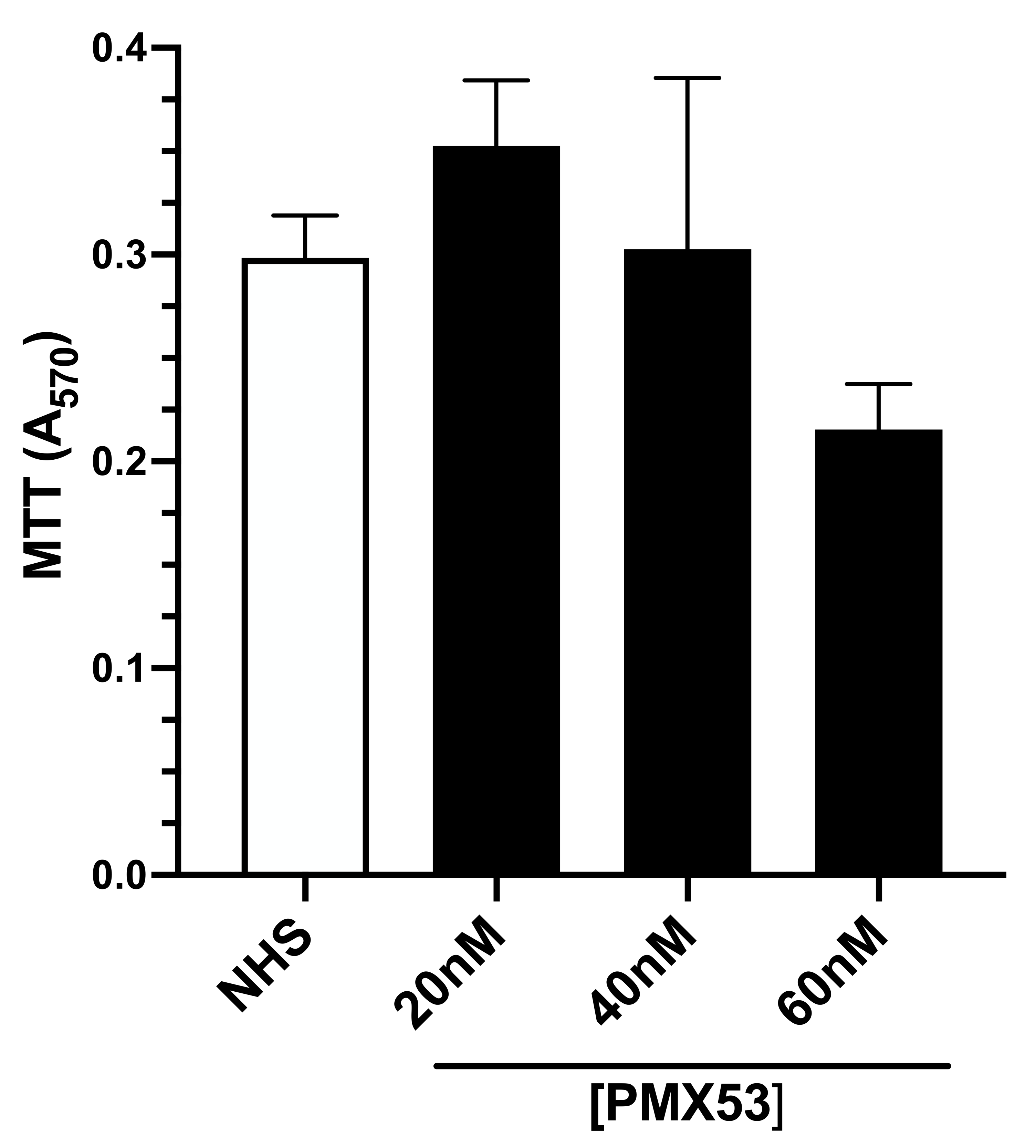
**
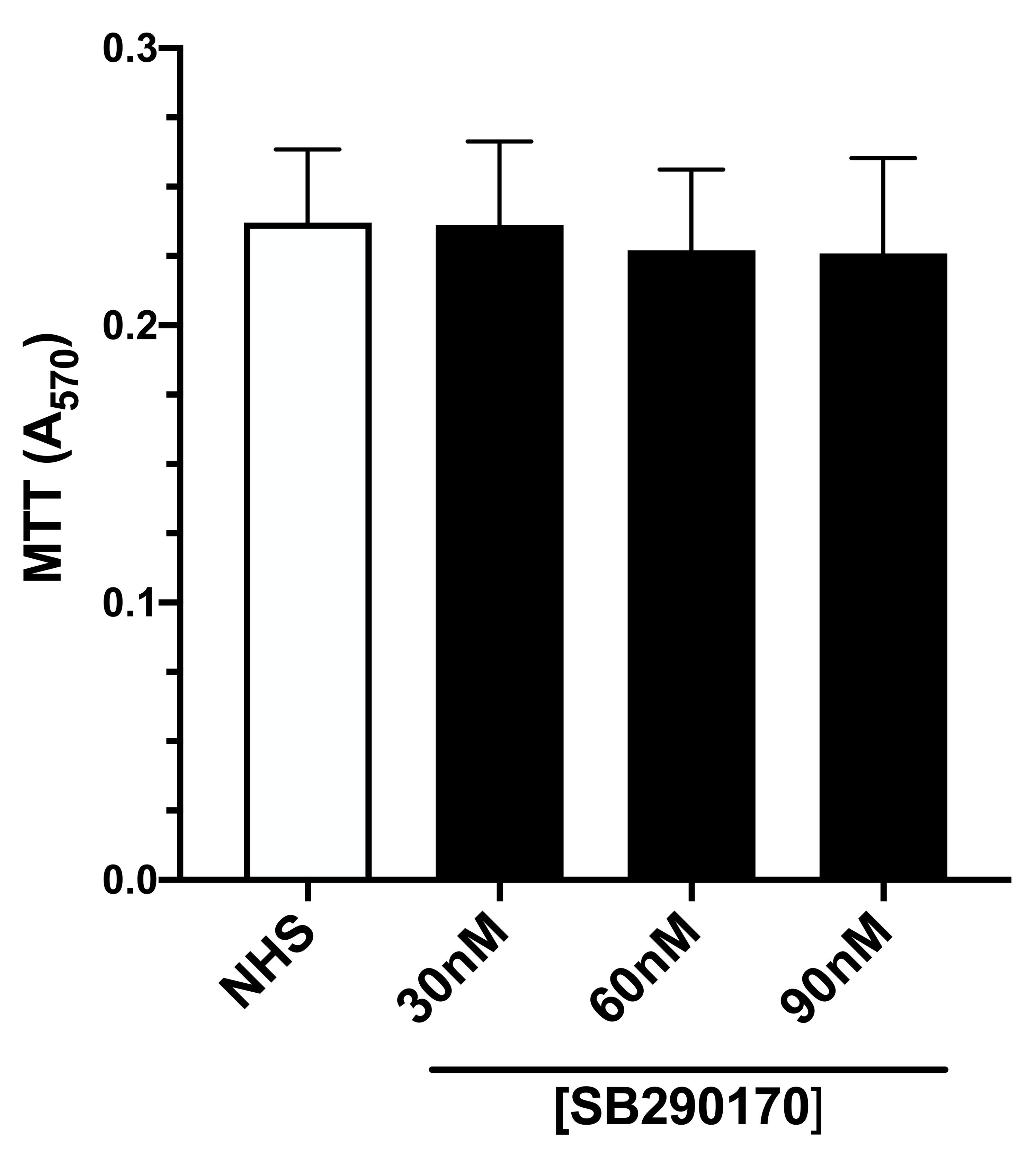
**A B**

**Table S1:** Primers used in the present study.

| Primers | 5’ to 3’ (Forward and Reverse) | length | Reference |
| --- | --- | --- | --- |
| *IL6* | GTAGCTCATTCTGCTCTGGAG  AGCATCAGTCCCAAGAAGGCAAC | 112 | This study |
| *IFNG* | CCTTTACTTCACTGACCAATAAG CCTGTTACTACCTGACACATTC | 120 | (1) |
| *NOS2* | ACCCGTCCACAGTATGTGAGG  CTCTGGGTCCTCTGGTCAAA | 154 | This study |
| *CD38* | GGTCCAAGTGATGCTCAATGGG  AGCTCCTTCGATGTCGTGCATC | 138 | This study |
| *C5LR2* | CTGCTGTCTACCGTAGGCTG AGAGGAATCGAACAGTGGTGA | 113 | (2) |
| *C3aR* | TCGATGCTGACACCAATTCAA  TCCCAATAGACAAGTGAGACCAA | 100 | (3) |
| *C5aR1* | CCATTAGTGCCGACCGTTTCCT  CACGAAGGATGGAATGGTGAGG | 133 | This study |
| *TNFA* | CACAGAAAGCATGATCCGCGAC  TGCCACAAGCAGGAATGAGAAGAG | 120 | (4) |
| Mouse *Actin* | CCTGTATGCCTCTGGTCGTA  CCATCTCCTGCTCGAAGTCT | 259 | (5) |
| Human *GADPH* | AGAAGGCTGGGGCTCATTTG  AGGGGCCATCCACAGTCTTC | 259 | (6) |
| *R. parkeri sca1* | TGCAGAGCAGTTGCTCG  CTAGCCTCGGAATGTTAGACC | 204 | This study |
| *R. rickettsii sca1* | TGCAGAGCAAGCTGCTCGTTATTACCC  CTAGCCTCGGAATGTTAGACC | 204 | This study |
| *R. australis sca1* | TGCAGAACAATTTGTTATTACC  CTACCGCTCCTTGGAACGTTAGACC | 215 | (7) |

**References:**

1. Gomez JA, Wapinski OL, Yang YW, Bureau JF, Gopinath S, Monack DM, Chang HY, Brahic M, Kirkegaard K. 2013. The NeST long ncRNA controls microbial susceptibility and epigenetic activation of the interferon-γ locus. Cell 152:743–754.

2. He Y, Yao X, Taylor N, Bai Y, Lovenberg T, Bhattacharya A. 2018. RNA sequencing analysis reveals quiescent microglia isolation methods from postnatal mouse brains and limitations of BV2 cells. J Neuroinflammation 15:1–13.

3. Litvinchuk A, Wan Y, Swartzlander DB, Zhang B, Litvinchuk A, Wan Y, Swartzlander DB, Chen F, Cole A, Propson NE. 2018. Complement C3aR inactivation attenuates Tau pathology and reverses an immune network deregulated in Tauopathy models and alzheimer’s Disease. Neuron 100:1337-1353.e5.

4. Deng Q, Sun M, Yang K, Zhu M, Chen K, Yuan J. 2013. MRP8 / 14 enhances corneal susceptibility to *Pseudomonas aeruginosa* infection by amplifying inflammatory responses. Invest Ophtalmol Vis Sci 54:3–5.

5. Riley SP, Fish AI, Garza DA, Banajee KH, Harris EK, del Piero F, Martinez JJ. 2016. Nonselective persistence of a *Rickettsia conorii* extrachromosomal plasmid during mammalian infection. Infect Immun 84:790–797.

6. Liu F, Fan H, Ren D, Dong G, Hu E. 2015. TLR9-induced miR-155 and Ets-1 decrease expression of CD1d on B cells in SLE. Eur J Immunol 45:1934–1945.

7. Riley SP, Fish AI, Del Piero F, Martinez JJ. 2018. Immunity against the obligate intracellular bacterial pathogen *Rickettsia australis* requires a functional complement system. Infect Immun 86:1–15.
